# Supplementary material for: Projecting COVID-19 isolation bed requirements for people experiencing homelessness
Source: PLoS One. 2021 May 12;16(5):e0251153. doi: 10.1371/journal.pone.0251153 (PMC8115830; doi:10.1371/journal.pone.0251153)

**S1** **Appendix. Sensitivity analyses for isolation bed demand estimates.**

**S1 Fig. Sensitivity analysis for proportion of COVID-19 cases that are tested (*p*(tested|infected)).** The red dashed line indicates the baseline value of 10% originally assumed. As this proportion tested increases, the projected maximum bed demand increases more rapidly under the four-week intervention scenario than under the four-month scenario.


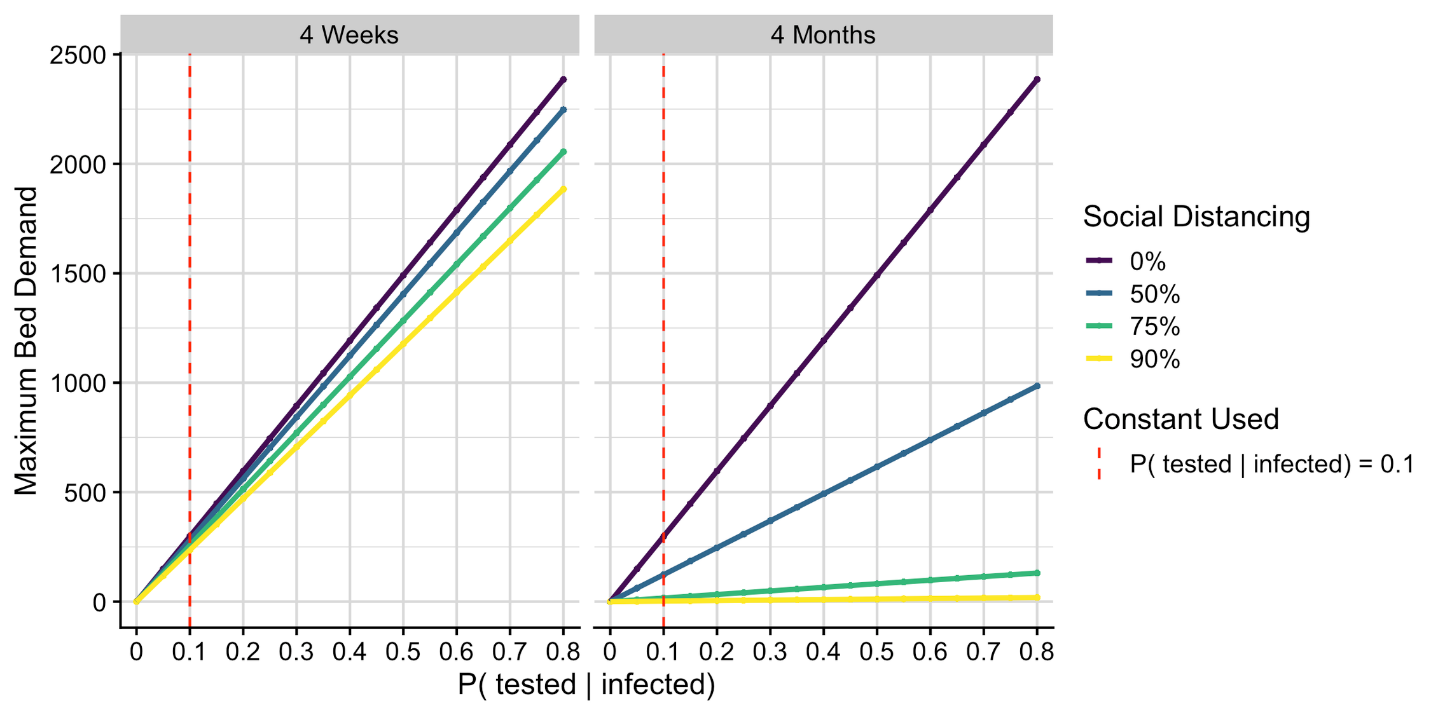


**S2 Fig. Sensitivity analysis for proportion of COVID-19 tests that are positive (*p*(infected|tested)).** The red dashed line indicates the baseline value of 9.8% originally assumed. As this test positivity increases, the projected maximum bed demand decreases more rapidly under the four-week intervention scenario than under the four-month scenario.


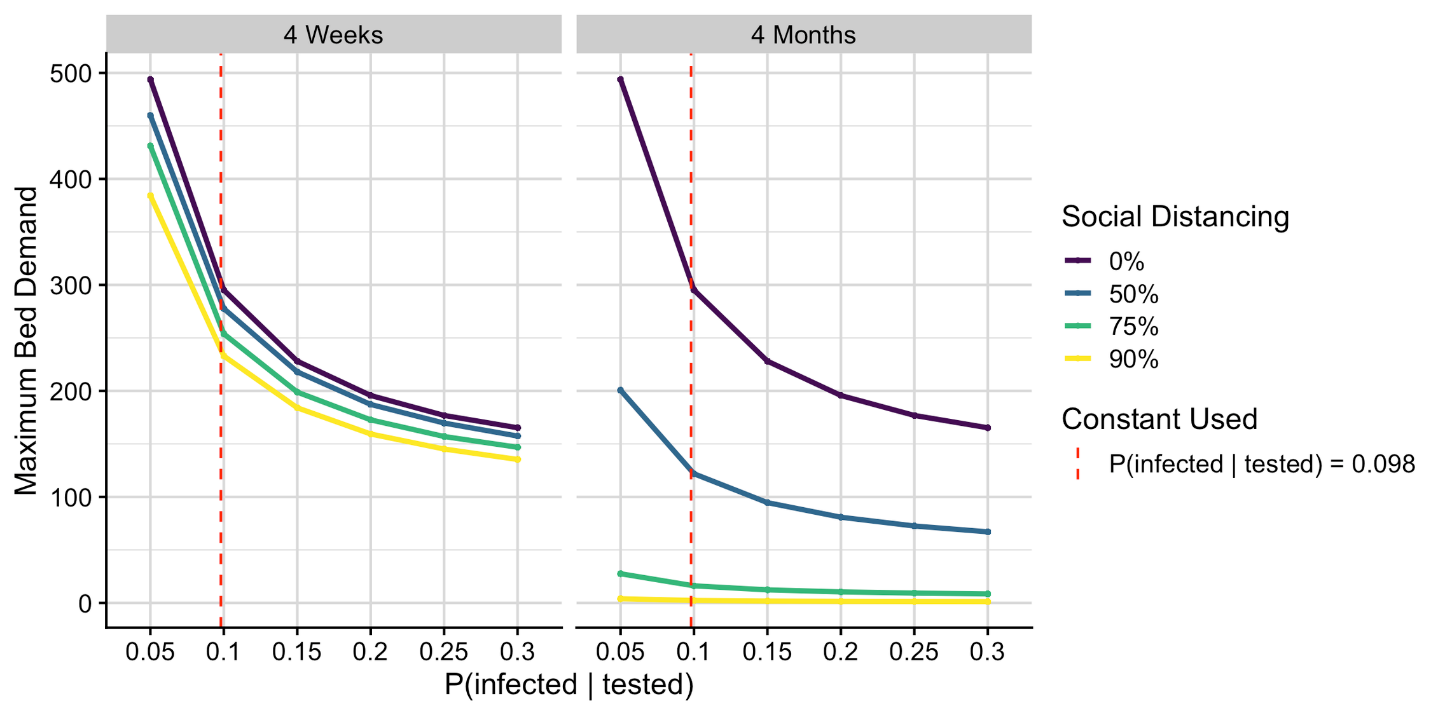


**S3 Fig. Sensitivity analysis for the delay between initial infection and testing (**[**
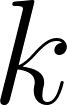
**](https://www.codecogs.com/eqnedit.php?latex=k#0)**).** The red dashed line indicates the baseline value of 5 days originally assumed. As [
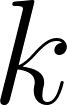
](https://www.codecogs.com/eqnedit.php?latex=k#0) increases, the expected peak demand stays flat under the four-week intervention scenario and decreases slightly under the four-month scenario. This decrease is an artifact of our decision to run simulations only until July 1st, capturing the first wave of the pandemic. As [
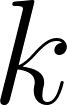
](https://www.codecogs.com/eqnedit.php?latex=k#0) increases, the curve shifts later into the summer, eventually past July 1st resulting in a smaller perceived peak bed demand. We note that our quick rule-of-thumb to calculate the maximum bed demand does not depend on this parameter.


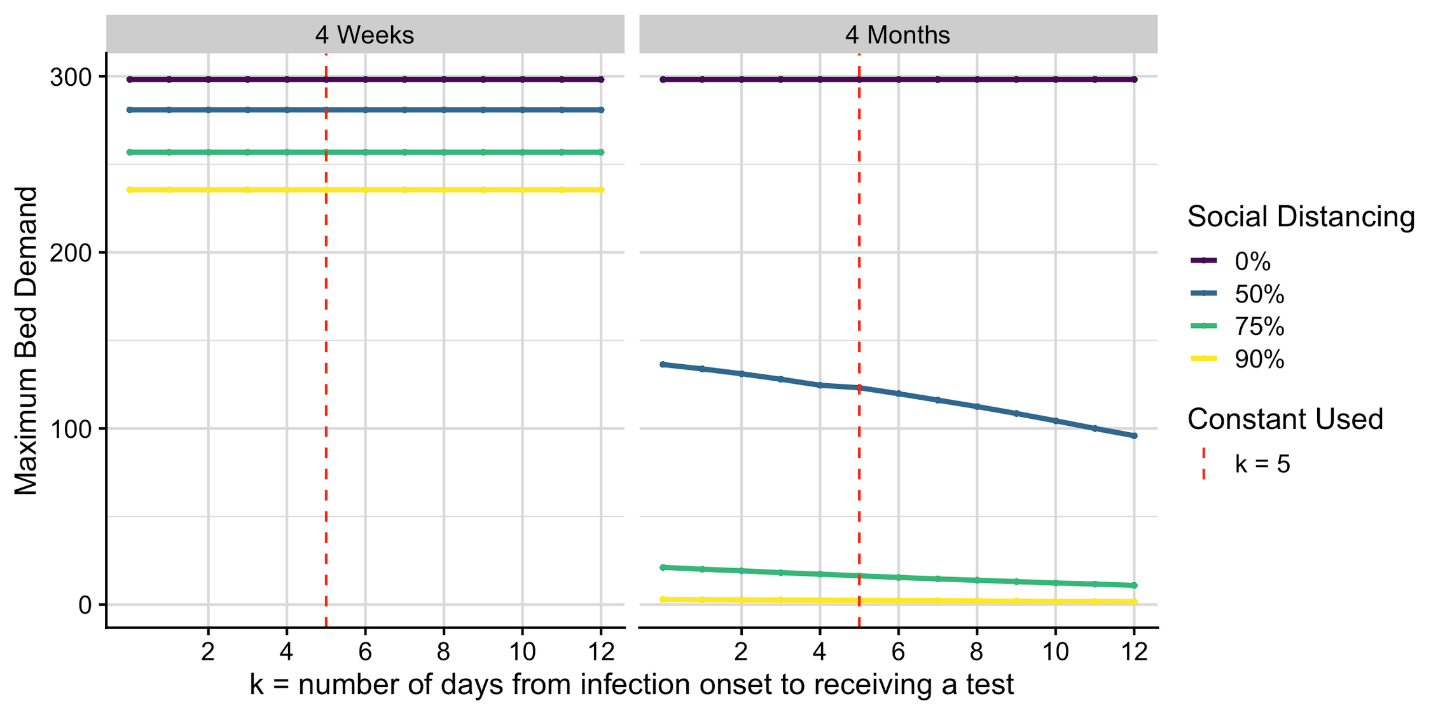


**S4 Fig. Sensitivity analysis for the number of days a positive case remains in isolation (**[**
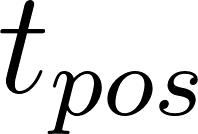
**](https://www.codecogs.com/eqnedit.php?latex=t_%7Bpos%7D#0)**).** The red dashed line indicates the baseline value of 14 days originally assumed. As [
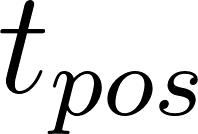
](https://www.codecogs.com/eqnedit.php?latex=t_%7Bpos%7D#0) increases, the expected peak demand increases more rapidly under the four-week intervention scenario than under the four-month scenario.


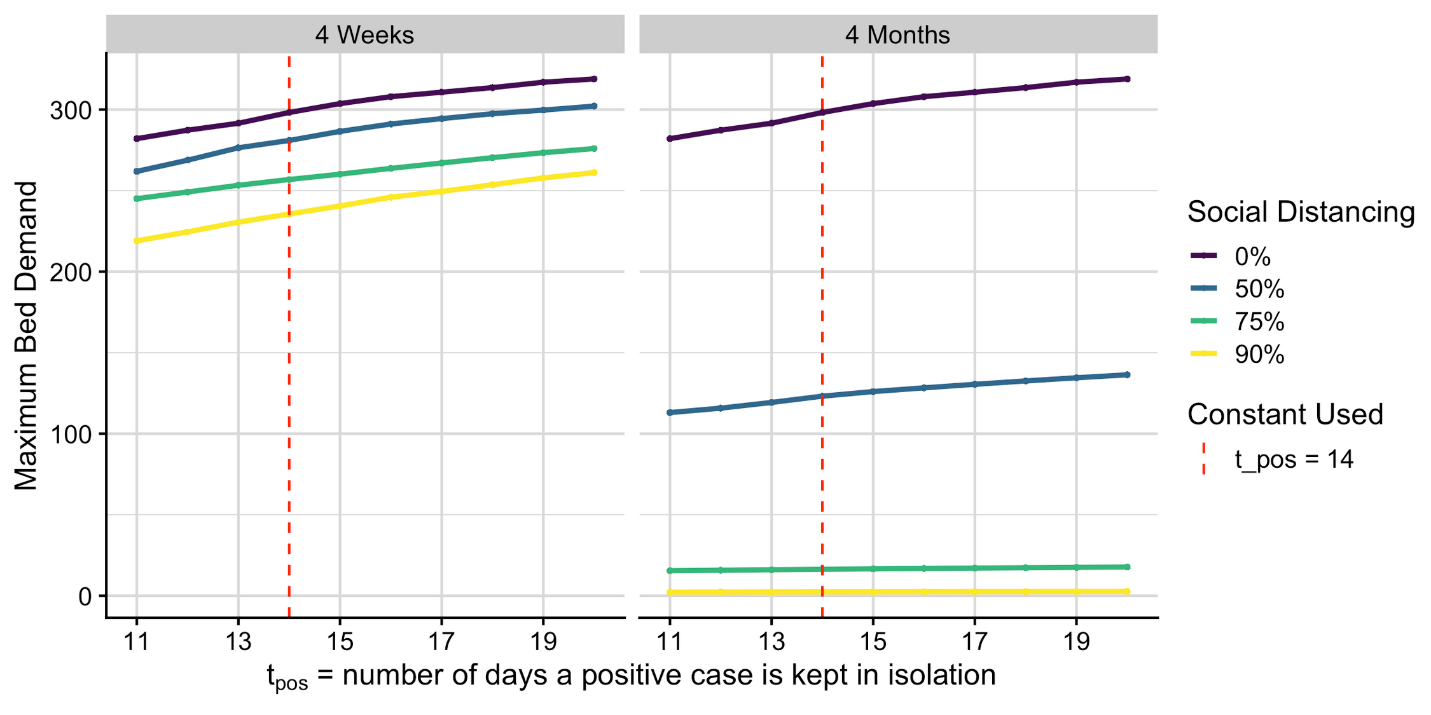


**S5 Fig. Sensitivity analysis for the number of days an individual who tests negative remains in isolation while waiting for their test result (** [**
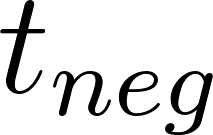
**](https://www.codecogs.com/eqnedit.php?latex=t_%7Bneg%7D#0)**).** The red dashed line indicates the baseline value of 2 days originally assumed. As [**
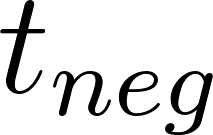
**](https://www.codecogs.com/eqnedit.php?latex=t_%7Bneg%7D#0) increases, the expected peak demand increases more rapidly under the four-week intervention scenario than under the four-month scenario.


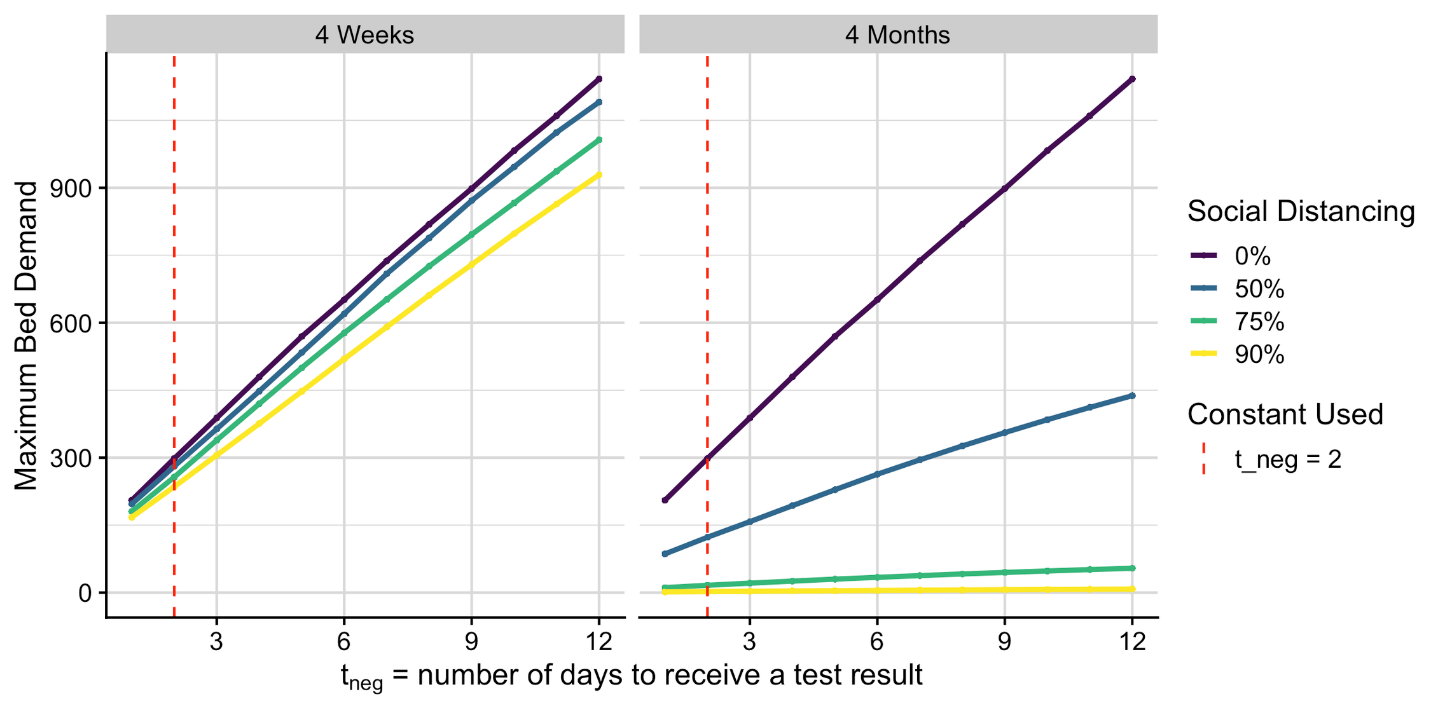

Supplement: S1 Appendix — (DOCX) [file pone.0251153.s001.docx]
