# Supplementary material for: Projecting COVID-19 isolation bed requirements for people experiencing homelessness
Source: PLoS One. 2021 May 12;16(5):e0251153. doi: 10.1371/journal.pone.0251153 (PMC8115830; doi:10.1371/journal.pone.0251153)
Supplement: S2 Appendix — (DOCX) [file pone.0251153.s002.docx]

## S2 Appendix. COVID-19 transmission model.

**S6 Fig. Compartmental model of COVID-19 transmission in a US city.** Each subgroup (defined by age and risk) is modeled with a separate set of compartments. Upon infection, susceptible individuals (S) progress to exposed (E) and then to either symptomatic infectious (I^Y^) or asymptomatic infectious (I^A^). All asymptomatic cases eventually progress to a recovered class where they remain protected from future infection (R); symptomatic cases are either hospitalized (I^H^) or recover. Mortality (D) varies by age group and risk group and is assumed to be preceded by hospitalization.


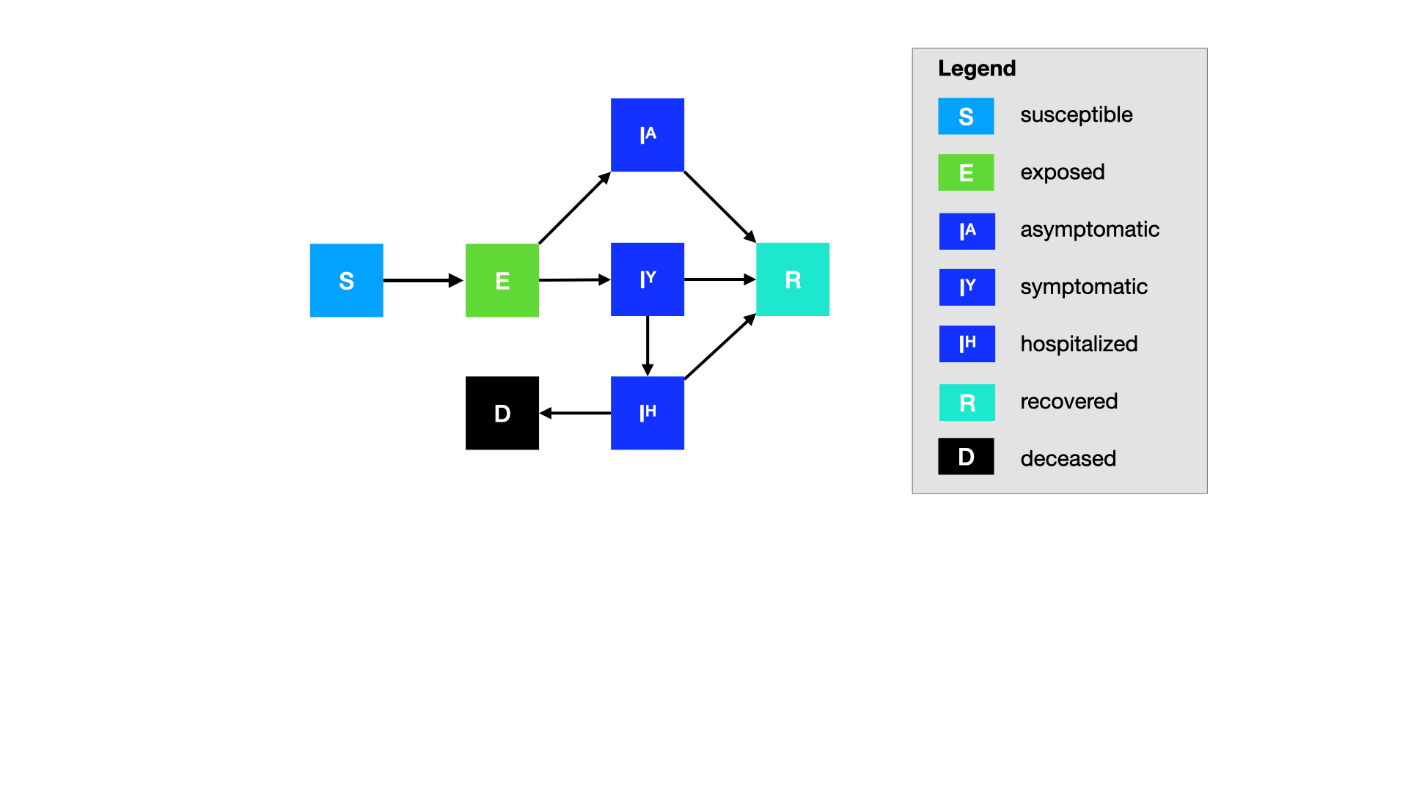


**S7 Fig. Comparing the relationship between peak COVID-19 cases and peak isolation bed requirements per 100 PEH in March 2020 and in July 2020.** For both of these timepoints in the pandemic, we fit 700 total stochastic simulations (100 per each of the 7 scenarios) to a linear model. In July 2020, where p(infected|tested) increased to 19.67% and p(tested|infected) increased to 20%, we found the linear model is [
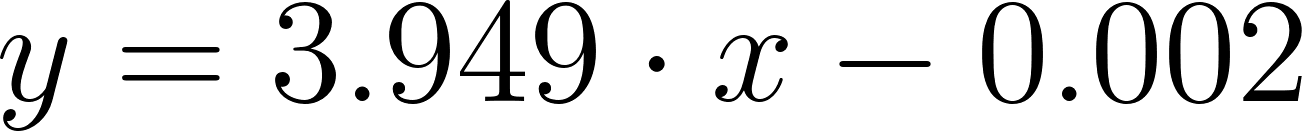
](https://www.codecogs.com/eqnedit.php?latex=y%20%3D%203.949%20%5Ccdot%20x%20-%200.002#0), where where [
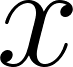
](https://www.codecogs.com/eqnedit.php?latex=x#0) is peak number of COVID-10 cases per 100 PEH and [
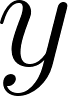
](https://www.codecogs.com/eqnedit.php?latex=y#0) is the peak bed requirement per 100 PEH (See Table S9 for regression table). This is roughly double the March 2020 model, [
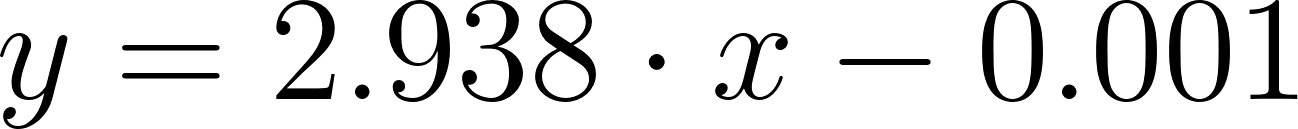
](https://www.codecogs.com/eqnedit.php?latex=y%20%3D%202.938%20%5Ccdot%20x%20-%200.001#0), where p(infected|tested) was 9.8% and p(tested|infected) was 10% (See Table S8 for regression table).


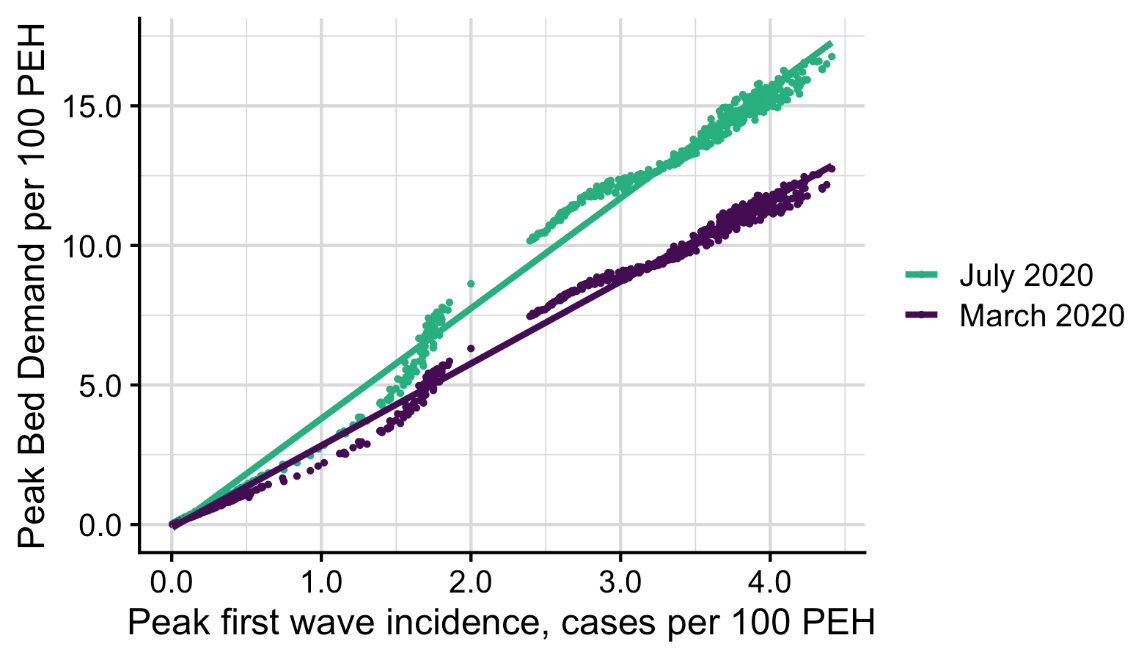


**S1 Table. Initial conditions, school closures and social distancing policies.**

| Variable | Settings |
| --- | --- |
| Initial day of simulation | 3/1/2020 |
| Initial infection number in locations | 5 symptomatic cases in 18-49y age group |
| Trigger to close school | 3/14/2020 |
| Closure Duration | Until start of 2020-2021 school year (8/17/20) |
| ɑ: Reduction of non-household contacts (work and other) | Five scenarios: [0, .25, 0.5, .75, 0.9] |
| Age-specific and day-specific contact rates | Home, work, other and school matrices provided in Tables S4-S7 in Appendix 2.  Normal weekday = home + work + other + school  Normal weekend = home + other  Normal weekday holiday = home + other  Normal weekday during summer or winter break = home + work + other  School closure weekday = home + (1-ɑ)*(work + other)  School closure weekend = home + (1-ɑ)*(other)  School closure weekday holiday = home + (1-ɑ)*(other)  School closure during summer or winter break = home + (1-ɑ)*(work + other) |

**S2 Table. Model parameters. Values given as five-element vectors are age-stratified with values corresponding to 0-4, 5-17, 18-49, 50-64, 65+ year age groups, respectively.** ^a^The parameter $\beta$ is fitted through constrained trust-region optimization in SciPy/Python. Given a value of $\beta$, a deterministic simulation is run based on central values for each parameter, from which we can compute the implied $\underline{R}_{0}\left( \beta\right)$. We (1) track the daily number of new cases $I_{t}$(both symptomatic and asymptomatic) during the exponential growth portion of the epidemic, (2) compute the log of the number of new cases: $y_{t}=log\left( I_{t} \right)$ and (3) use least squares to fit a line to this curve: $loglog \left( I_{t} \right) =y_{0}+g\cdot t$. We then estimate the reproduction number $\underline{R}_{0}\left( \beta\right)$ of the simulation for that specific value of $\beta$ as $\underline{R_{0}}\left( \beta\right)=\Gamma\cdot g+$ 1 where $\Gamma$is the generation time given by $\Gamma=\frac{\delta\left( R_{0}-1 \right)}{loglog \left( 2 \right)}$. The optimizing function runs until the resulting value of $\underline{R}_{0}\left( \beta\right)$ does not get closer to the target value.

| Parameters | Best guess - values (doubling time = 7.2 days) | Best guess values  (doubling time = 4 days) | Source |
| --- | --- | --- | --- |
| R_0_ | 2.2 | 2.2 | Li et al. [(2)](https://paperpile.com/c/WhMEE0/TcDn9) |
| $\delta$: doubling time | 7.2 days | 4 days | Kraemer et al. [(3)](https://paperpile.com/c/WhMEE0/TOsV7) |
| $\beta$: transmission rate | 0.01622242 | 0.02599555 | Fitted^a^ to obtain specified R_0_ given $\delta$ |
| $\gamma^{A}$: recovery rate on asymptomatic compartment | Equal to $\gamma^{Y}$ | |  |
| $\gamma^{Y}$: recovery rate on symptomatic non-treated compartment | $\frac{1}{\gamma^{Y}}\sim Triangular\left( 21.2,22.6,24.4 \right)$ | | Verity et al. (4) |
| $\tau$: symptomatic proportion (%) | 82.1 | | Mizumoto et al. [(5)](https://paperpile.com/c/WhMEE0/7HmAY) |
| $\sigma$: exposed rate | $\frac{1}{\sigma}\sim Triangular\left( 5.6,7,8.2 \right)$ | | Lauer et al. [(6)](https://paperpile.com/c/WhMEE0/V6hPW) |
| P: proportion of pre-symptomatic (%) | 12.6 | | Du et al. (7) |
| $\omega^{E}$: relative infectiousness of infectious individuals in compartment E | $\omega^{E}=\frac{\left( \frac{YHR}{\eta}+\frac{1-YHR}{\gamma^{Y}} \right)\omega^{Y}\sigma P}{1-P}$ | |  |
| $\omega^{A}$: relative infectiousness of infectious individuals in compartment I^A^ | 0.4653 | | Set to mean of [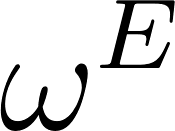](https://www.codecogs.com/eqnedit.php?latex=%5Comega%5EE#0) |
| IFR: infected fatality ratio, age specific (%) | Overall: [0.0016, 0.0049, 0.084, 1.000, 3.371]  Low risk: [0.00091668, 0.0021789, 0.03388, 0.25197, 0.64402]  High risk: [0.009167, 0.02179, 0.33878, 2.5197, 6.4402] | | Age adjusted from Verity et al. [(4)](https://paperpile.com/c/WhMEE0/ULIBS) |
| YFR: symptomatic fatality ratio, age specific (%) | Overall: [0.001949, 0.006025, 0.10265, 1.2182, 4.10657]  Low risk: [0.0011165, 0.002654, 0.04126, 0.3069, 0.78443]  High risk: [0.01117, 0.02654, 0.4126, 3.06903, 7.8443] | | $YFR=\frac{IFR}{1-\tau}$ |
| $h$: high-risk proportion, age specific (%) | [8.2825, 14.1121, 16.5298, 32.9912, 47.0568] | | Estimated using 2015-2016 Behavioral Risk Factor Surveillance System (BRFSS) data with multilevel regression and poststratification using CDC’s list of conditions that may increase the risk of serious complications from influenza (8-10) |
| $rr$: relative risk for high risk people compared to low risk in their age group | 10 | | Assumption |
| School calendars | Austin Independent School District calendar (2019-2020, 2020-2021) | | [(11)](https://paperpile.com/c/WhMEE0/rsbiG) |

**References:**

1. [minimize(method=’trust-constr’) — SciPy v1.4.1 Reference Guide [Internet]. [cited 2020 Mar 28]. Available from:](http://paperpile.com/b/WhMEE0/zuAGg) <https://docs.scipy.org/doc/scipy/reference/optimize.minimize-trustconstr.html>
2. [Li Q, Guan X, Wu P, Wang X, Zhou L, Tong Y, et al. Early Transmission Dynamics in Wuhan, China, of Novel Coronavirus-Infected Pneumonia. N Engl J Med. 2020 Mar 26;382(13):1199–207.](http://paperpile.com/b/WhMEE0/TcDn9)
3. [Kraemer MUG, Yang C-H, Gutierrez B, Wu C-H, Klein B, Pigott DM, et al. The effect of human mobility and control measures on the COVID-19 epidemic in China. medRxiv [Internet]. 2020; Available from:](http://paperpile.com/b/WhMEE0/TOsV7) <https://www.medrxiv.org/content/10.1101/2020.03.02.20026708v1>
4. [Verity R, Okell LC, Dorigatti I, Winskill P, Whittaker C, Imai N, et al. Estimates of the severity of COVID-19 disease [Internet]. Epidemiology. medRxiv; 2020. Available from:](http://paperpile.com/b/WhMEE0/ULIBS) <https://www.medrxiv.org/content/10.1101/2020.03.09.20033357v1.abstract>
5. [Mizumoto K, Kagaya K, Zarebski A, Chowell G. Estimating the Asymptomatic Proportion of 2019 Novel Coronavirus onboard the Princess Cruises Ship, 2020 [Internet]. Infectious Diseases (except HIV/AIDS). medRxiv; 2020. Available from:](http://paperpile.com/b/WhMEE0/7HmAY) <https://www.medrxiv.org/content/10.1101/2020.02.20.20025866v2>
6. [Lauer SA, Grantz KH, Bi Q, Jones FK, Zheng Q, Meredith HR, et al. The Incubation Period of Coronavirus Disease 2019 (COVID-19) From Publicly Reported Confirmed Cases: Estimation and Application. Ann Intern Med [Internet]. 2020 Mar 10; Available from:](http://paperpile.com/b/WhMEE0/V6hPW) <http://dx.doi.org/10.7326/M20-0504>
7. [Du Z, Xu X, Wu Y, Wang L, Cowling BJ, Meyers LA. The serial interval of COVID-19 from publicly reported confirmed cases [Internet]. Epidemiology. medRxiv; 2020. Available from:](http://paperpile.com/b/WhMEE0/VKDMO) <https://www.medrxiv.org/content/10.1101/2020.02.19.20025452v3>
8. [CDC. People at High Risk of Flu [Internet]. Centers for Disease Control and Prevention. 2019 [cited 2020 Mar 26]. Available from:](http://paperpile.com/b/WhMEE0/DPoP2) <https://www.cdc.gov/flu/highrisk/index.htm>
9. [CDC - BRFSS [Internet]. 2019 [cited 2020 Mar 26]. Available from:](http://paperpile.com/b/WhMEE0/55bW5) <https://www.cdc.gov/brfss/index.html>
10. [Zhang X, Holt JB, Lu H, Wheaton AG, Ford ES, Greenlund KJ, et al. Multilevel regression and poststratification for small-area estimation of population health outcomes: a case study of chronic obstructive pulmonary disease prevalence using the behavioral risk factor surveillance system. Am J Epidemiol. 2014 Apr 15;179(8):1025–33.](http://paperpile.com/b/WhMEE0/tMLJF)
11. [Calendar of Events [Internet]. Austin ISD. [cited 2020 Mar 26]. Available from:](http://paperpile.com/b/WhMEE0/rsbiG) <https://www.austinisd.org/calendar>

**S3 Table. Hospitalization parameters.**

| Parameters | Value | Source |
| --- | --- | --- |
| $\gamma^{H}$: recovery rate in hospitalized compartment | 0.0869565 | 11.5 day-average from admission to discharge [(1)](https://paperpile.com/c/WhMEE0/rV2Gr) |
| YHR: symptomatic case hospitalization rate (%) | Overall: [ 0.04872107, 0.04872107, 3.28757227, 11.33739519, 17.73306336]  Low risk: [0.0279, 0.0215, 1.3215, 2.8563, 3.3873]  High risk: [ 0.2791, 0.2146, 13.2154, 28.5634, 33.8733] | Age adjusted from Verity et al. [(2)](https://paperpile.com/c/WhMEE0/ULIBS) |
| $\pi$: rate of symptomatic individuals go to hospital, age-specific | $\pi=\frac{\gamma^{Y}\cdot YHR}{\eta+\left( \gamma^{Y}-\eta\right)YHR}$ |  |
| $\eta$: rate from symptom onset to hospitalized | 0.1695 | 5.9 day average from symptom onset to hospital admission Tindale et al. [(3)](https://paperpile.com/c/WhMEE0/8XAvC) |
| $\mu$: rate from hospitalized to death | 0.0892857 | 11.2 day-average from admission to death [(1)](https://paperpile.com/c/WhMEE0/rV2Gr) |
| HFR: hospitalized fatality ratio, age specific (%) | [4, 12.365, 3.122, 10.745, 23.158] | $HFR=\frac{IFR}{YHR\left( 1-\tau\right)}$ |
| $\nu$: death rate on hospitalized individuals, age specific | [0.0390, 0.1208, 0.0304, 0.1049, 0.2269] | $\nu=\frac{\gamma^{H}HFR}{\mu+\left( \gamma^{H}-\mu\right)HFR}$ |
| ICU: proportion hospitalized people in ICU | [0.15, 0.20, 0.15, 0.20, 0.15] | CDC COVID-19 planning scenarios (based on US seasonal flu data) |
| Vent: proportion of individuals in ICU needing ventilation | [0.35, 0.3, 0.45, 0.5, 0.45] | CDC planning scenarios  (based on US seasonal flu data) |
| $d_{ICU}$: duration of stay in ICU | 8 days | Assumption, computed as average of hospital stay and ventilation durations |
| $d_{V}$: duration of ventilation | 5 days | CDC COVID-19 planning scenarios |
| $HCS$: healthcare capacity | Hospital bed: 4299  ICU bed: 755  Ventilator: 755 | Estimates provided by each of the region's hospital systems and aggregated by regional public health leaders |

**References:**

1. [Sanche S, Lin YT, Xu C, Romero-Severson E, Hengartner N, Ke R. The Novel Coronavirus, 2019-nCoV, is Highly Contagious and More Infectious Than Initially Estimated [Internet]. Epidemiology. medRxiv; 2020. Available from:](http://paperpile.com/b/WhMEE0/rV2Gr) <http://dx.doi.org/10.1101/2020.02.07.20021154>
2. [Verity R, Okell LC, Dorigatti I, Winskill P, Whittaker C, Imai N, et al. Estimates of the severity of COVID-19 disease [Internet]. Epidemiology. medRxiv; 2020. Available from:](http://paperpile.com/b/WhMEE0/ULIBS) <https://www.medrxiv.org/content/10.1101/2020.03.09.20033357v1.abstract>
3. [Tindale L, Coombe M, Stockdale JE, Garlock E, Lau WYV, Saraswat M, et al. Transmission interval estimates suggest pre-symptomatic spread of COVID-19 [Internet]. Epidemiology. medRxiv; 2020. Available from:](http://paperpile.com/b/WhMEE0/8XAvC) <http://dx.doi.org/10.1101/2020.03.03.20029983>

**S4 Table. Home contact matrix (daily number contacts by age group at home).**

|  | 0-4y | 5-17y | 18-49y | 50-64y | 65y+ |
| --- | --- | --- | --- | --- | --- |
| 0-4y | 0.5 | 0.9 | 2.0 | 0.1 | 0.0 |
| 5-17y | 0.2 | 1.7 | 1.9 | 0.2 | 0.0 |
| 18-49y | 0.2 | 0.9 | 1.7 | 0.2 | 0.0 |
| 50-64y | 0.2 | 0.7 | 1.2 | 1.0 | 0.1 |
| 65y+ | 0.1 | 0.7 | 1.0 | 0.3 | 0.6 |

**S5 Table. School contact matrix (daily number contacts by age group at school).**

|  | 0-4y | 5-17y | 18-49y | 50-64y | 65y+ |
| --- | --- | --- | --- | --- | --- |
| 0-4y | 1.0 | 0.5 | 0.4 | 0.1 | 0.0 |
| 5-17y | 0.2 | 3.7 | 0.9 | 0.1 | 0.0 |
| 18-49y | 0.0 | 0.7 | 0.8 | 0.0 | 0.0 |
| 50-64y | 0.1 | 0.8 | 0.5 | 0.1 | 0.0 |
| 65y+ | 0.0 | 0.0 | 0.1 | 0.0 | 0.0 |

**S6 Table. Work contact matrix (daily number contacts by age group at work).**

|  | 0-4y | 5-17y | 18-49y | 50-64y | 65y+ |
| --- | --- | --- | --- | --- | --- |
| 0-4y | 0.0 | 0.0 | 0.0 | 0.0 | 0.0 |
| 5-17y | 0.0 | 0.1 | 0.4 | 0.0 | 0.0 |
| 18-49y | 0.0 | 0.2 | 4.5 | 0.8 | 0.0 |
| 50-64y | 0.0 | 0.1 | 2.8 | 0.9 | 0.0 |
| 65y+ | 0.0 | 0.0 | 0.1 | 0.0 | 0.0 |

**S7 Table. Others contact matrix (daily number contacts by age group at other locations).**

|  | 0-4y | 5-17y | 18-49y | 50-64y | 65y+ |
| --- | --- | --- | --- | --- | --- |
| 0-4y | 0.7 | 0.7 | 1.8 | 0.6 | 0.3 |
| 5-17y | 0.2 | 2.6 | 2.1 | 0.4 | 0.2 |
| 18-49y | 0.1 | 0.7 | 3.3 | 0.6 | 0.2 |
| 50-64y | 0.1 | 0.3 | 2.2 | 1.1 | 0.4 |
| 65y+ | 0.0 | 0.2 | 1.3 | 0.8 | 0.6 |

**S8 Table. Regression table for linear model estimating peak bed requirement from peak COVID-19 incidence in PEH under parameters from March 2020.** Here we set p(tested | infected) as 10% and p(infected | tested) as 9.8%.

| *Predictors* | *Estimates* | *95% CI* |
| --- | --- | --- |
| (Intercept) | -0.001 | -0.002, -0.001 |
| Peak Incidence | 2.938 | 2.923, 2.954 |
| Observations | 700 |  |
| R^2^ / R^2^ Adjusted | 0.995 / 0.995 |  |

**S9 Table. Regression table for linear model estimating peak bed requirement from peak COVID-19 incidence in PEH under parameters from July 2020.** Here we set p(tested | infected) as 20% and p(infected | tested) as 19.67%.

| *Predictors* | *Estimates* | *95% CI* |
| --- | --- | --- |
| (Intercept) | -0.002 | -0.002, -0.001 |
| Peak Incidence | 3.949 | 3.925, 3.973 |
| Observations | 700 |  |
| R^2^ / R^2^ Adjusted | 0.993 / 0.993 |  |

**S10 Table. Comparing projected cumulative hospitalizations and peak daily COVID-19 hospital admissions to observed data from the City of Austin.**

| Duration of Intervention | Contact Reduction | Projected Cumulative Hospitalizations | Peak Daily COVID-19 Hospital Admissions |
| --- | --- | --- | --- |
| No Intervention | 0% | 76,246 (61,598 - 91,030) | 10,892 (8,800 - 13,004) |
| 4 Weeks | 50% | 75,393 (60,757 - 91,932) | 10,770 (8,680 - 13,133) |
|  | 75% | 75,948 (61,223 - 91,129) | 10,850 (8,746 - 13,018) |
|  | 90% | 76,127 (60,684 - 91,696) | 10,875 (8,669 - 13,099) |
| 4 Months | 50% | 68,984 (54,971 - 83,562) | 9,855 (7,853 - 11,937) |
|  | 75% | 1,971 (1,223 - 3,532) | 282 (175 - 505) |
|  | 90% | 444 (265 - 774) | 63 (38 - 111) |
| Observed  Austin Data | -- | 1,685 | 78 |
